# Supplementary material for: A modular self-adjuvanting cancer vaccine combined with an oncolytic vaccine induces potent antitumor immunity
Source: Nat Commun. 2021 Aug 31;12:5195. doi: 10.1038/s41467-021-25506-6 (PMC8408233; doi:10.1038/s41467-021-25506-6)
Supplement: Supplementary file 1 — Supplementary Information [file 41467_2021_25506_MOESM1_ESM.pdf]

Supplementary Materials

A modular self-adjuvanting cancer vaccine combined with an oncolytic vaccine induces potent antitumor immunity

Das et al.

- Supplementary Figure 1
- Supplementary Figure 2
- Supplementary Figure 3
- Supplementary Figure 4
- Supplementary Figure 5
- Supplementary Figure 6
- Supplementary Figure 7
- Supplementary Figure 8
- Supplementary Figure 9
- Supplementary Figure 10
- Supplementary Figure 11
- Supplementary Figure 12
- Supplementary Figure 13

- Supplementary Table 1
- Supplementary Table 2
- Supplementary Table 3
- Supplementary Table 4
- Supplementary Table 5
- Supplementary Table 6
- Supplementary Table 7
- Supplementary Table 8
- Supplementary Table 9

Corresponding authors:

guido.wollmann@i-med.ac.at
madiha.derouazi@boehringer-ingelheim.com

Supplementary Figure 1

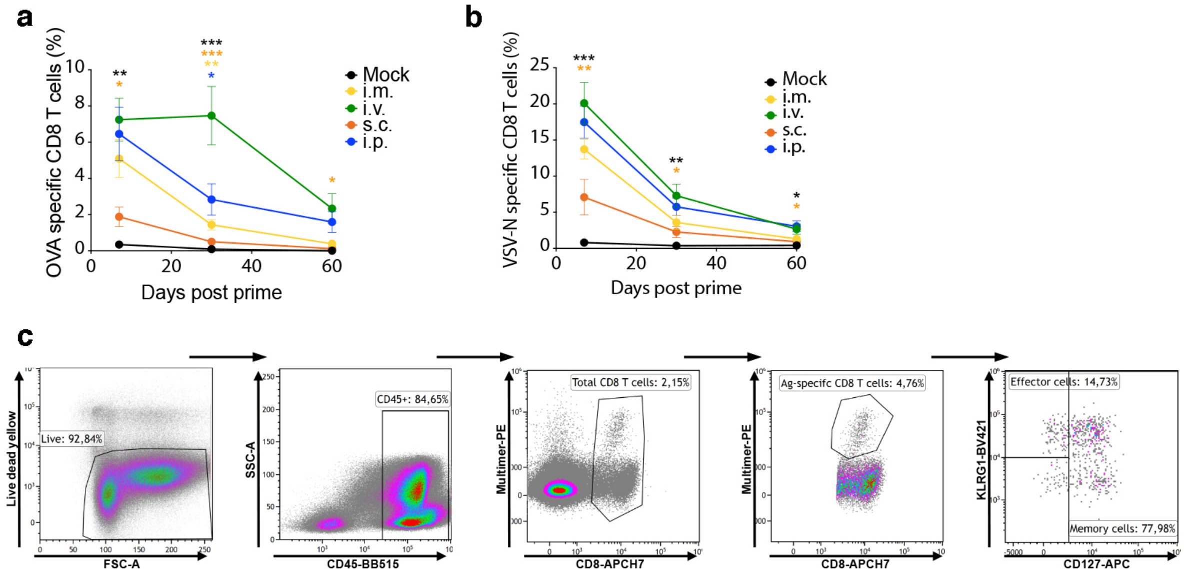

Supplementary Fig. 1. Systemic delivery of VSV-GP-OVA is highly immunogenic. a, b Mice received a single dose of $1x10^7$ $TCID_{50}$ VSV-GP-OVA given i.m., i.v., s.c. or i.p (n=5 per treatment group, n=3 for mock). Proportion of **a** OVA- and **b** VSV-N-specific $CD8^+$ T cells in the periphery on days 7, 30 and 60 post prime is depicted (mean ± SEM). One-way ANOVA with Tukey's multiple comparisons (*, p<0.05; **, p< 0.01; ***, p< 0.001; asterisks shown in reference to i.v. group). **c** Gating strategy for flow cytometric analysis of antigen-specific $CD8^+$ T cells for memory ($CD127^+$) and effector ($KLRG\text{-}1^+CD127^-$) cell subsets. This study was performed once. The comparison of i.m. vs i.v. application was repeated once. Source data and p-values are provided in the Source Data File.

Supplementary Figure 2

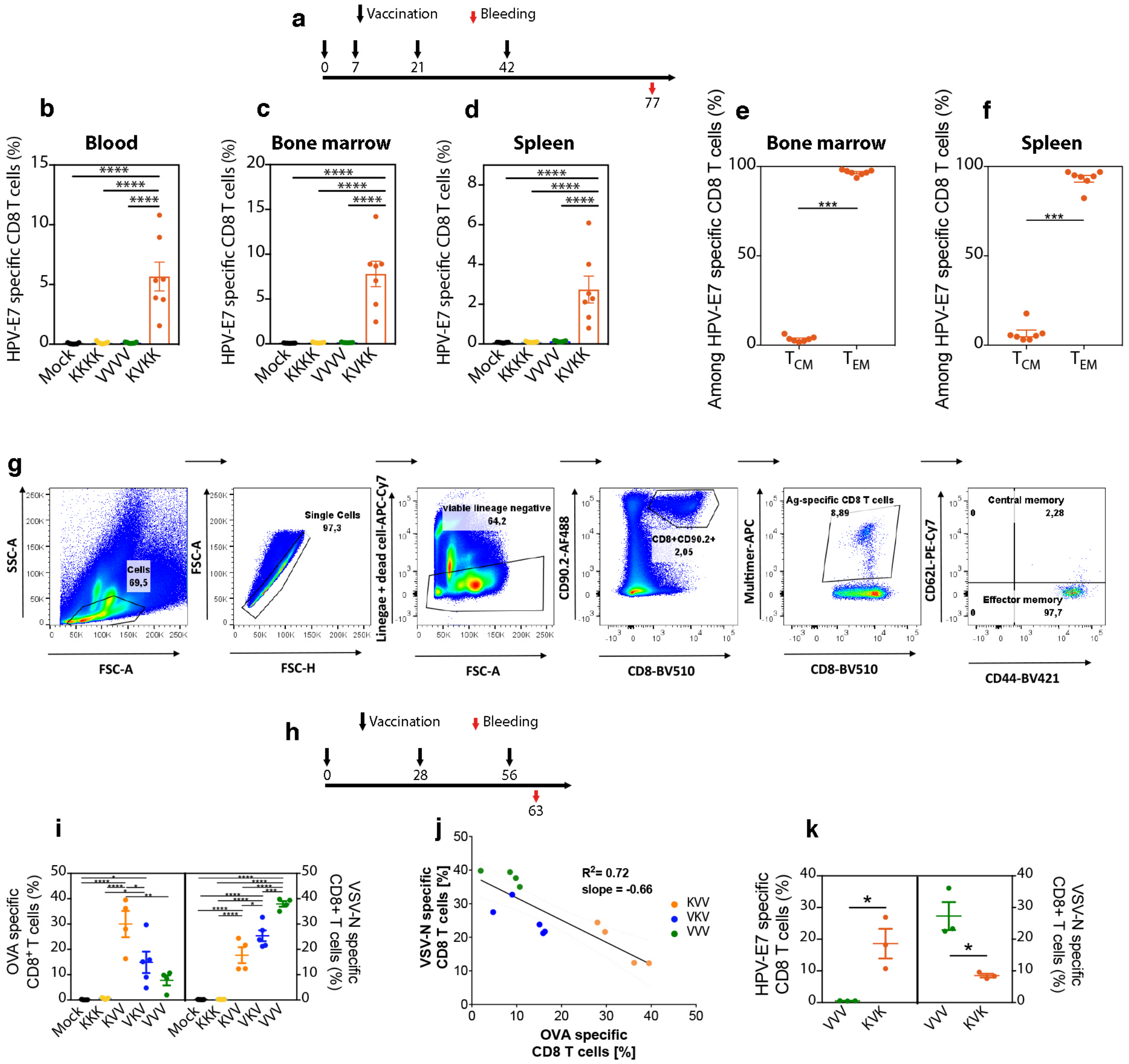

Supplementary Fig. 2. Heterologous prime-boost with KISIMA-TAA and VSV-GP-TAA promotes long-lasting antitumor immunity. a-g C57BL/6J mice were immunized with KISIMA-HPV (2 nmol, s.c.) or with VSV-GP-HPV (1x10^7 $TCID_{50}$, i.v.) on days 0, 7, 21 and 42 (n=7 per treatment group). HPV-E7-specific $CD8^+$ T cells in blood, spleen and bone barrow (BM) were analyzed 5 weeks after last immunization as indicated in schematic **a.** **b-d** The persistence of E7-specific T cells in **b** circulation, **c** bone marrow and **d** spleen is shown (mean ± SEM). **e, f** The distribution of central memory (T_{CM}) and effector memory (T_{EM}) cells among E7-specific $CD8^+$ T cells in **e** bone marrow and **f** spleen of mice receiving heterologous vaccination (KVKK) is graphed. Two-tailed Mann-Whitney test (***, p< 0.001). **g** Gating strategy for defining effector and central memory cells among antigen-specific $CD8^+$ T cells. Non-viable cells and cells from other lineages (including CD4, CD19 and CD14) were excluded. One-way ANOVA with Tukey's multiple comparisons was performed for comparison between different groups (****p<0.0001). **h-j** C57BL/6J mice were immunized with KISIMA-OVA (2 nmol, s.c.) or with VSV-GP-OVA (1x10^7 $TCID_{50}$, i.m.) on days 0, 28 and 56. Blood was drawn for flow cytometric analysis 7 days post 3rd vaccination as indicated in schematic **h.** **i** The proportion of OVA- and VSV-N-specific $CD8^+$ T cells is depicted (n=5 for mock, KKK, and VKV, n= 4 for KVV

and VVV). One-way ANOVA with Tukey's multiple comparison (*, $p < 0.05$; ****$p < 0.0001$). **j** Correlation between the frequency of OVA- and VSV-N-specific $CD8^+$ T cells in blood for various vaccine regimens is shown (linear regression analysis). **k** C57BL/6J mice were immunized with KISIMA-HPV (2 nmol, s.c.) or with VSV-GP-HPV (1×10^7 $TCID_{50}$, i.v.) on days 0, 7 and 21 (n=3 per treatment group). The frequencies of circulating E7- and VSV-N-specific $CD8^+$ T cells on day 28 is shown (mean ± SEM). Two-tailed unpaired t test (*, $p < 0.05$). Studies **b – i** were performed once. Study **k** was repeated three times. Source data and p-values are provided in the Source Data File.

Supplementary Figure 3

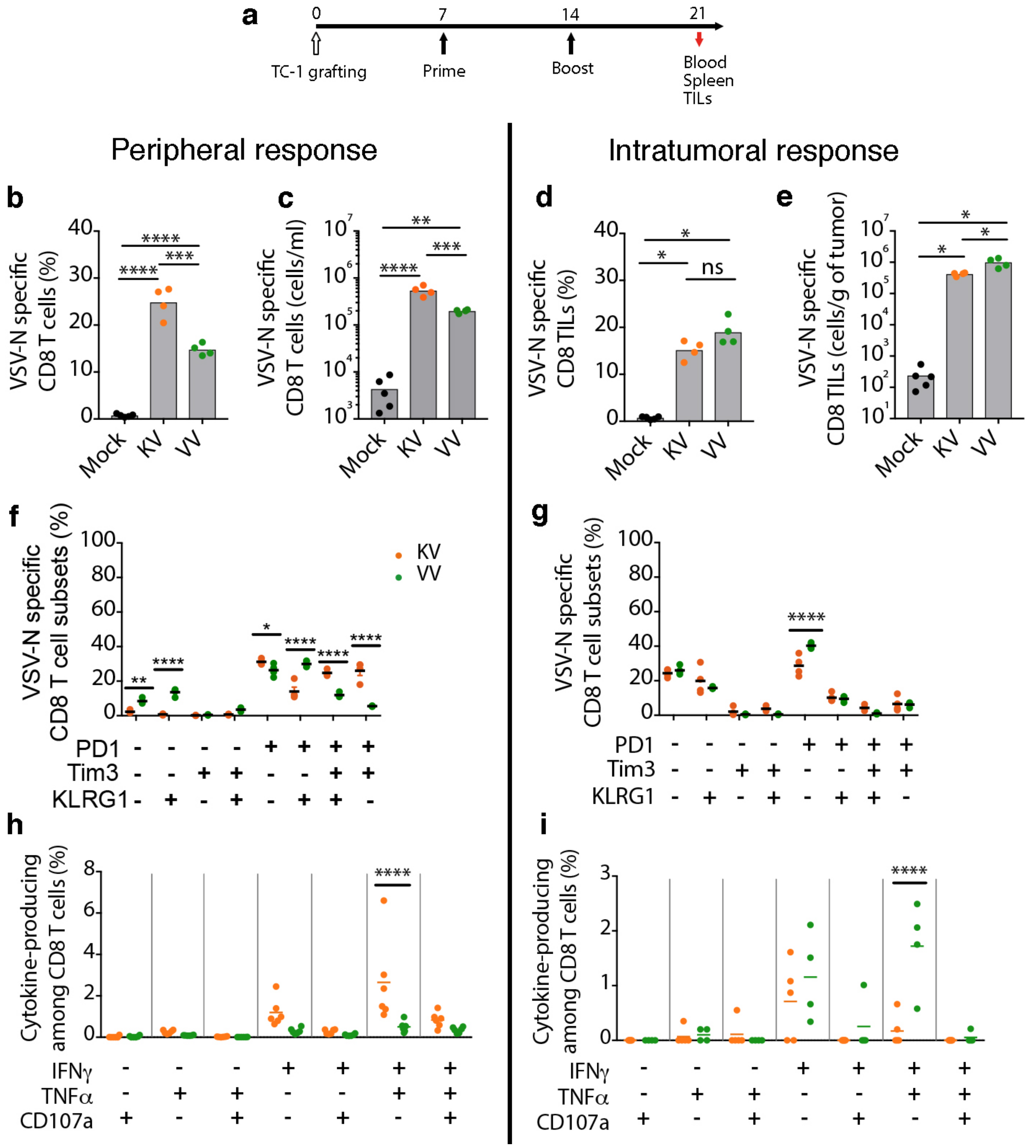

Supplementary Fig. 3. Functionality of peripheral and intratumoral VSV-specific CTLs. a-i C57BL/6J mice were injected s.c. with TC-1 cells ($1x10^5$) on day 0 and vaccinated with KISIMA-HPV (2 nmol, s.c.) or VSV-GP-HPV ($1x10^7$ $TCID_{50}$, i.v.) on day 7 and 14. Blood, spleen and tumors were harvested on day 21 for flow cytometric analysis (n=5 for mock, n= 4 for each treatment group). **a** Schematic of experimental plan. **b, d** Frequencies and **c, e** numbers of VSV-N-specific $CD8^+$ T cells in **b, c** blood (two-tailed one-way ANOVA with Tukey's multiple comparisons was performed with ***p<0.001; ****p<0.0001) and in **d, e** tumors (Mann-Whitney test with * p<0.05) are shown. **f, g** Proportions of **f** peripheral and **g** intratumoral VSV-N-specific $CD8^+$ T cells expressing activation and exhaustion markers are depicted. 2-way ANOVA with Sidak's multiple comparisons (****, p<0.0001). **h, i** Frequencies of VSV-N-specific $CD8^+$ T cells secreting different cytokines following *ex vivo*

restimulation among **h** splenic (n=5 for mock and KV, n= 6 for VV) or **i** intratumoral (n=5 for mock, n= 4 for each treatment group) $CD8^+$ T cells are shown. 2-way ANOVA with Tukey's multiple comparisons was performed (****p<0.0001). All data shown as individual data points and mean. This study was performed once. Source data and p-values are provided in the Source Data File.

Supplementary Figure 4

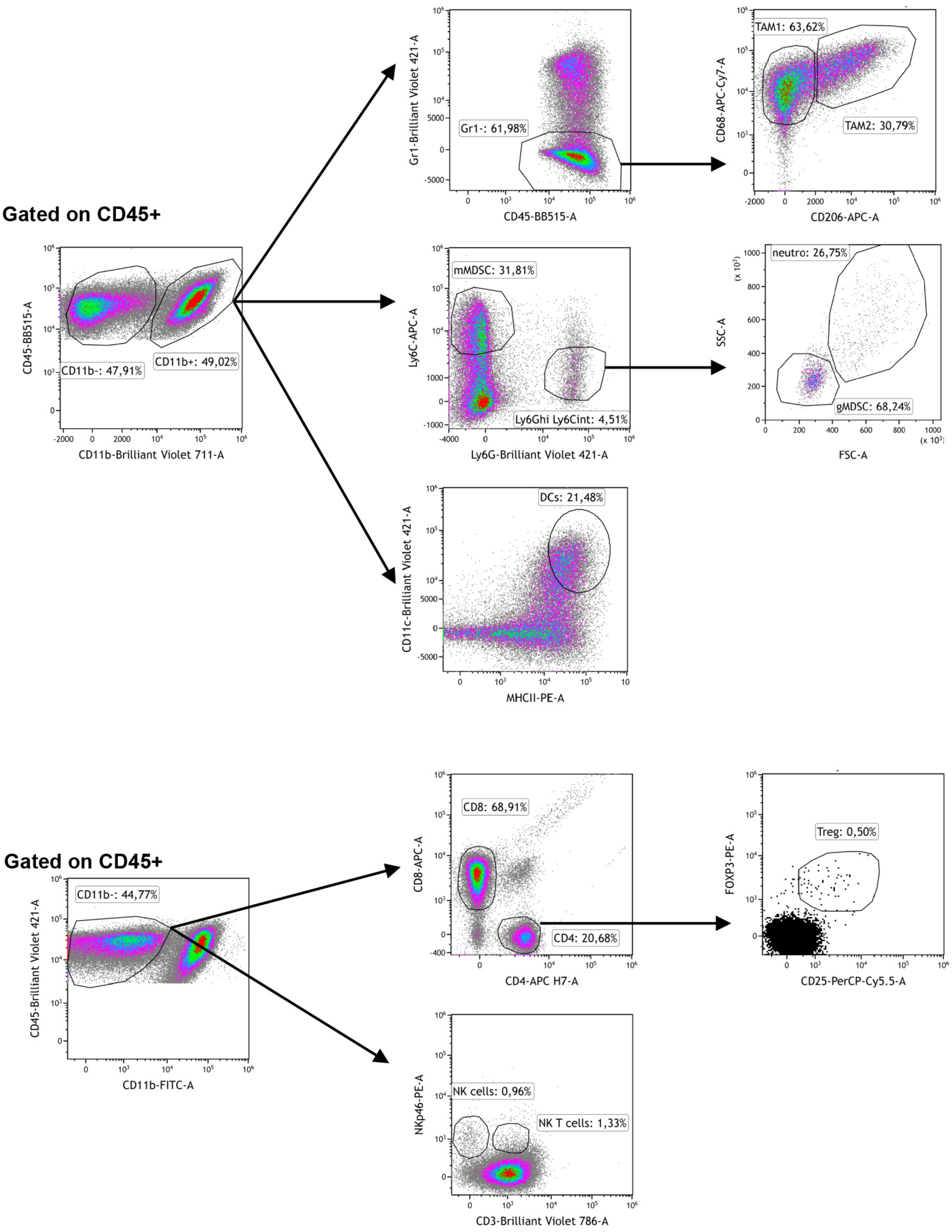

Supplementary Fig. 4. Flow cytometry gating strategy applied for the determination of various tumor-infiltrating leukocyte subpopulations described in Fig. 4. Cells were gated based on forward- and side-scatter characteristics, doublets were excluded and then gated on all $CD45^+$ events.

Supplementary Figure 5

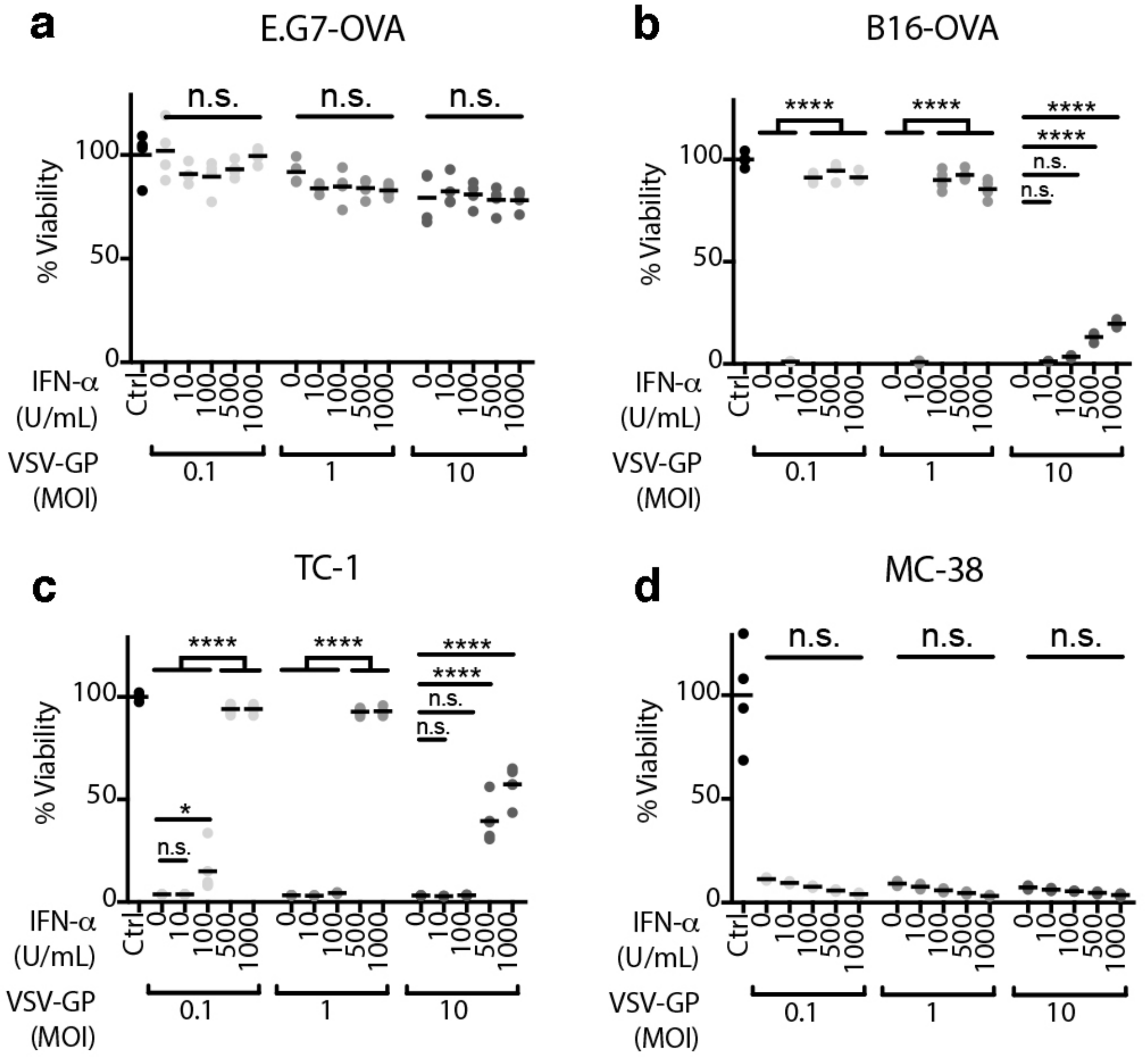

Supplementary Fig. 5. Different tumor models display different levels of susceptibility to VSV-GP mediated oncolysis and responsiveness to type I interferons *in vitro*. Cell viability of **a** E.G7-OVA **,** **b** B16-OVA, **c** TC-1 and **d** MC38 cells upon VSV-GP infection at increasing doses (MOI 0.1,1,10) and IFN-α pre-treatment (0–1000 U/mL overnight) was measured 72 h post infection using MTT assay. All data shown as individual data points and mean (n = 4). One-way ANOVA with Tukey's multiple comparisons (*, p<0.05; **, p<0.01; ***, p<0.001; ****, p<0.0001, n.s. not significant). The studies **a – d** have been performed independently three times. Source data and p-values are provided in the Source Data File.

Supplementary Figure 6

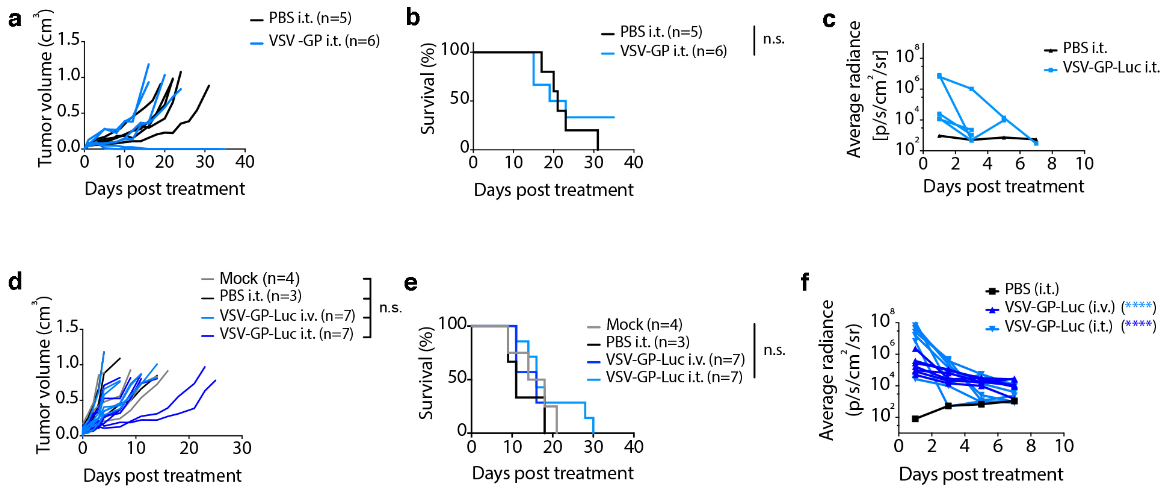

Supplementary fig. 6. Intratumoral replication of VSV-GP-Luc in different syngeneic tumor models. a-i C57BL/6J mice were subcutaneously implanted with **a-c** MC38 ($2x10^5$) or **d-f** TC-1 ($1.5x10^5$) cells and tumors were treated at a median tumor size of 0.05-0.07 cm^3 with a single dose of $1x10^8$ $TCID_{50}$ VSV-GP-Luc or PBS injected either intratumorally (i.t) or intravenously (i.v.) as indicated. **a, d** Individual tumor growth kinetics and **b, e** survival of the animals are depicted. Pair-wise Log-rank test was performed. **c, f** The activity of luciferase was measured as a surrogate of viral load using the *in vivo* bioluminescence imaging (BLI) system IVIS. The quantification of the average radiance in the tumour area is depicted for individual tumors over time. One-way ANOVA with Tukey's multiple comparisons (****, p<0.0001, n.s. not significant). The study shown in **a – c** has been performed once. The study shown in **d – f** has been performed two times. Source data and p-values are provided in the Source Data File.

Supplementary Figure 7

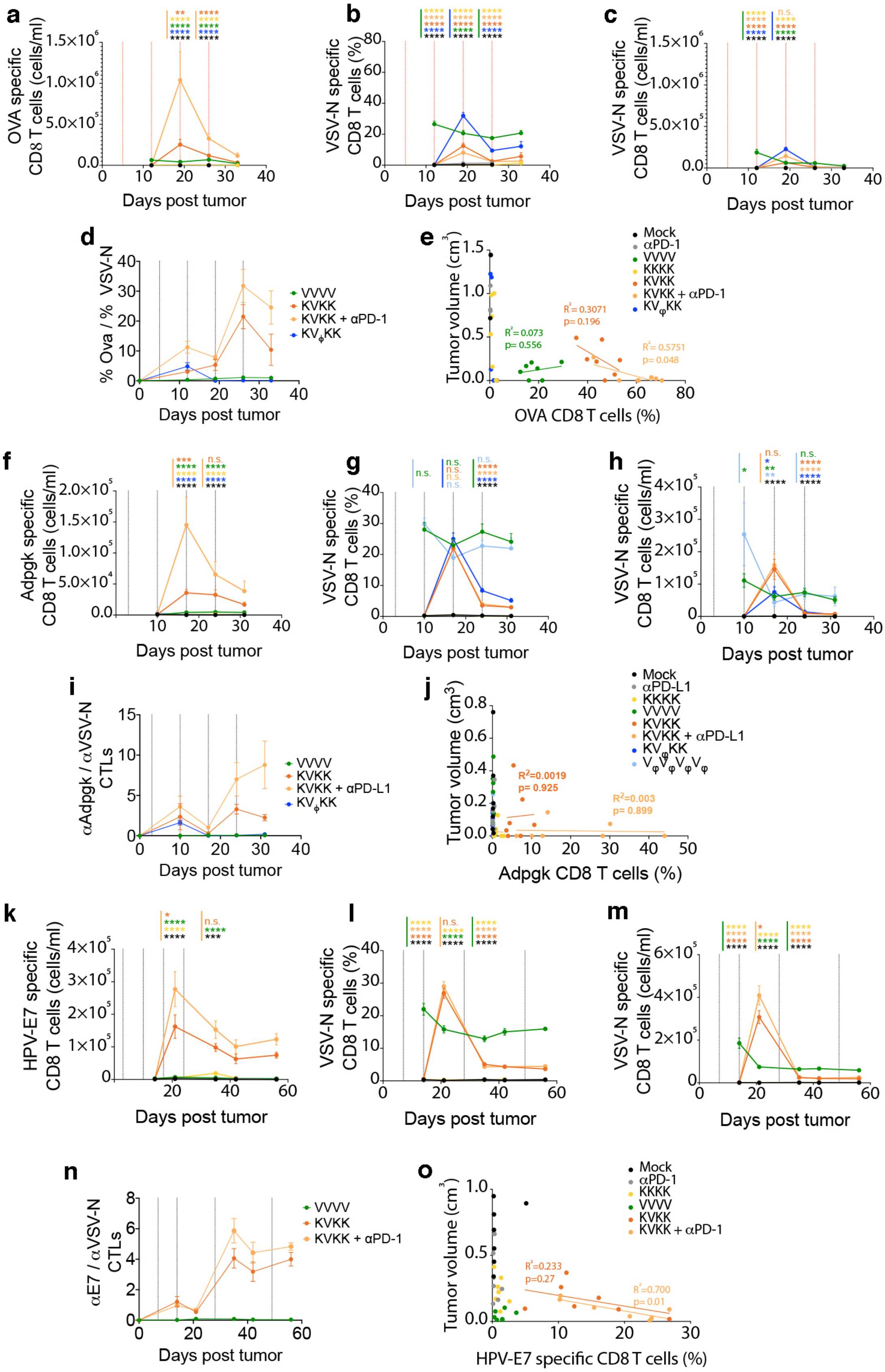

Supplementary Fig. 7. **Clonal expansion of tumor antigen-specific T cells in periphery after heterologous prime-boost vaccination in tumor-bearing mice is further enhanced by checkpoint blockade.** Mice bearing **a-e** E.G7-OVA (n=7), **f-j** MC38 (n=5 for $KV_{\phi}KK$ and n=7 for every other treatment group) and **k-o** TC-1 (n=7) tumors were immunized against the respective tumor-antigen using KISIMA-TAA (K) or the relevant VSV-GP-TAA as in Fig. 5 (E.G7-OVA) and Fig. 6 (MC-38 and TC-1). Anti-tumor and anti-viral $CD8^+$ T cells in the periphery were assessed 7 days post each immunization. **a, f, k** The number of **a** OVA-, **f** Adpgk- and **k** E7-specific $CD8^+$ T cells per ml blood is depicted (mean ± SEM). **b, g, l** The proportion and **c, h, m** number (cells/ml) of anti-viral $CD8^+$ T cells in circulation is shown (mean ± SEM). **d, i, n** The ratio of anti-tumor (**d** Ova, **i** Adpgk and **n** E7) to anti-viral CD8+ T cells is depicted (mean ± SEM). **e, j, o** The correlation between tumor-specific $CD8^+$ T cell frequencies 7 days post 2nd immunization and tumor size is graphed for **e** OVA-specific $CD8^+$ T cells and EG.7-OVA tumors, **j** Adpgk-specific $CD8^+$ T cells and MC38 tumors and **o** E7–specific $CD8^+$ T cells and TC-1 tumors. One way ANOVA with Tukey's multiple comparison was performed and statistical significance displayed only for tumor-specific T cells in KVKK treatment group compared to KVKK and checkpoint blockade treatment group (*p<0.05; **p<0.01; ***p<0.001; ****p<0.0001; colored asterisks indicate group analysis in reference to groups with respective highest amplitude, which is indicated by the vertical colored line). Linear regression analysis performed for testing correlation. The studies **a-j** have been performed once. The study depicted in **i-m** has been repeated once. Source data and p-values are provided in the Source Data File.

Supplementary Figure 8

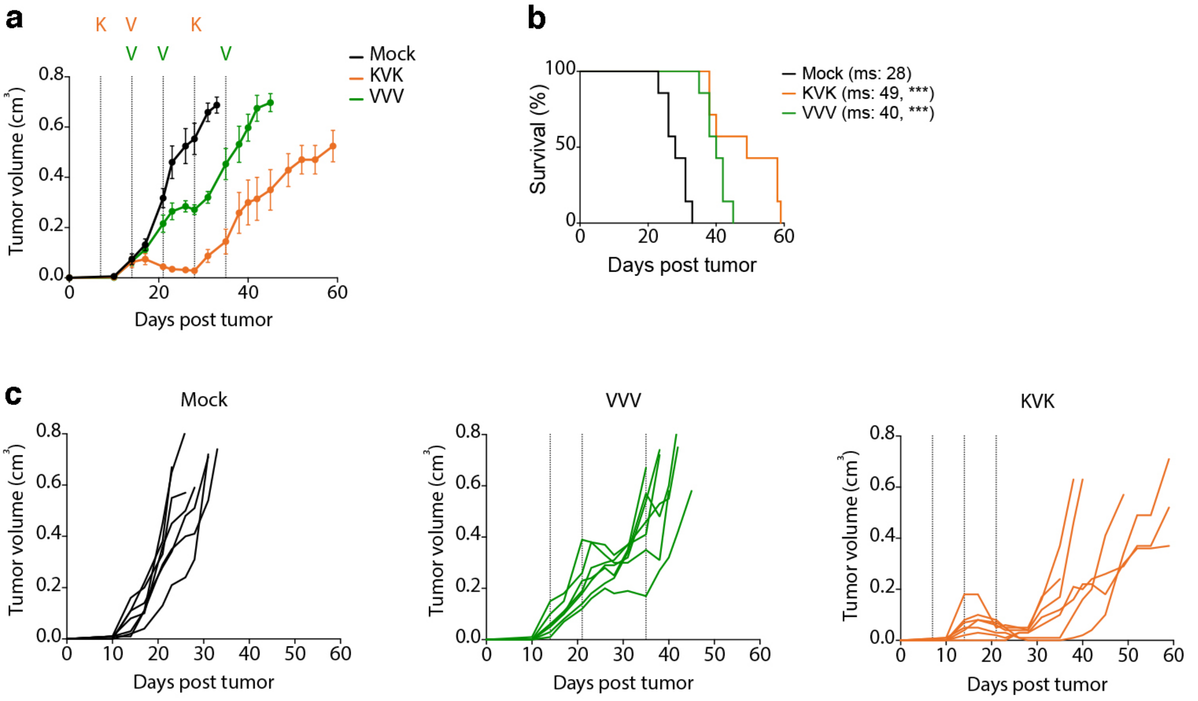

Supplementary Fig. 8. KISIMA-HPV prime is crucial for the therapeutic effect of heterologous vaccination in TC-1 tumor model. a-c Mice were injected with $1x10^5$ TC-1 cells s.c. and were immunized with 2 nmol KISIMA s.c. 7 days later or with $1x10^7$ $TCID_{50}$ VSV-GP-HPV i.v. 14 days post tumor implantation. Additional doses of K and V were administered as indicated by dotted lines and tumor growth was monitored (n=7). **a** Tumor growth (mean ± SEM), **b** survival and **c** individual tumor growth is depicted. Pairwise Log-rank test was performed (***p<0.001). This study was performed once. Source data and p-values are provided in the Source Data File.

Supplementary Figure 9

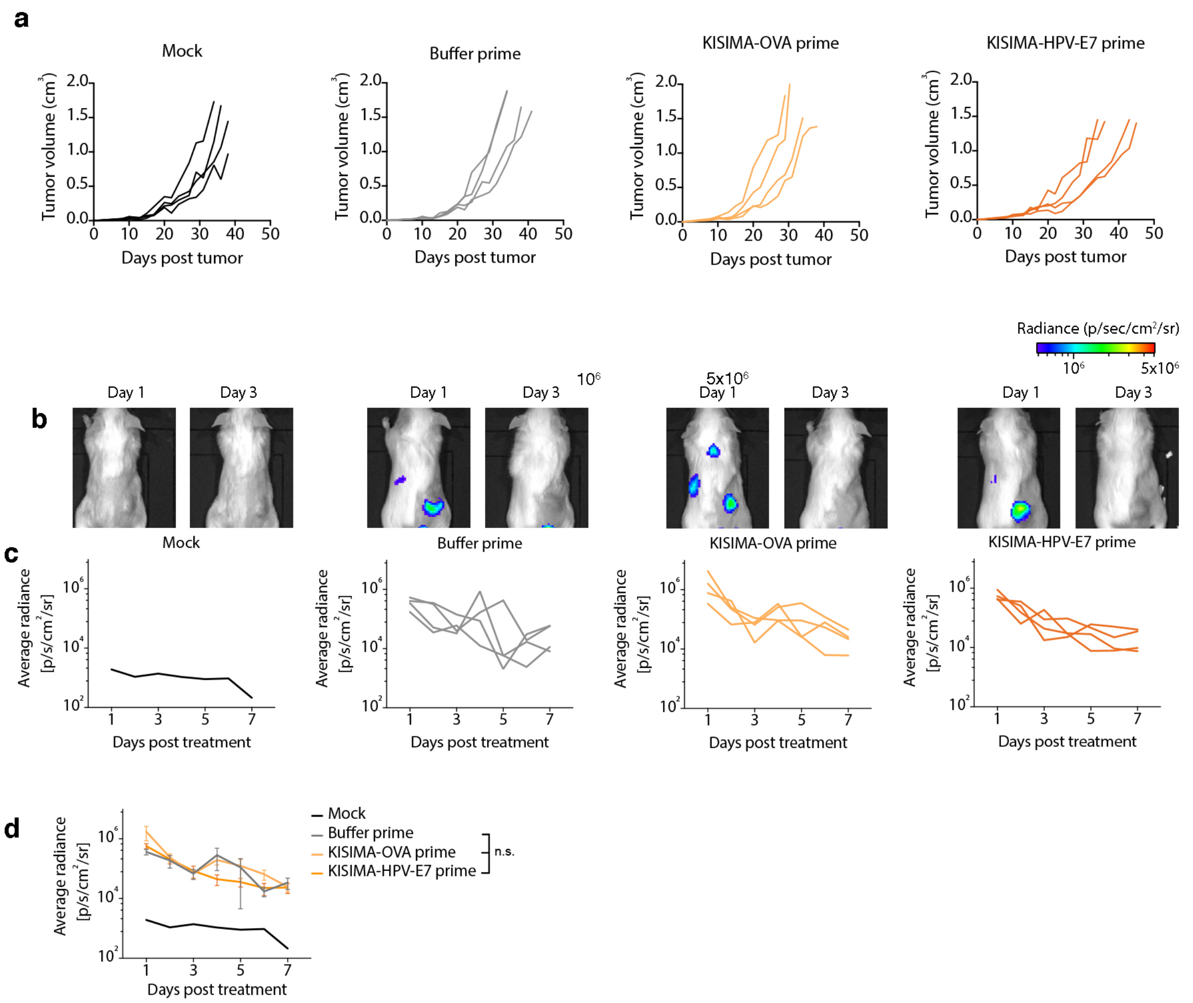

Supplementary Fig. 9. KISIMA-HPV prime does not enhance replication of VSV-GP in TC-1 tumors. a- d B6(C)/Rj-Tyrc/c (albino) mice were injected with $1x10^5$ TC-1 cells s.c. followed s.c. immunization with 2 nmol KISIMA-HPV (specific for TC-1 tumors), KISIMA-OVA (unrelated vaccine), buffer only or left untreated (mock) 7 days later. On day 14 post tumor implantation, mice received a single i.v. injection of $1x10^8$ $TCID_{50}$ VSV-GP-Luc (n=4). **a** Tumor growth, **b** exemplary bioluminescence images, **c** bioluminescence of individual animals is depicted and **d** average bioluminescence signal (mean ± SEM) are shown. One-way ANOVA with Tukey's multiple comparisons (ns: not significant). The study as shown was performed once. Source data are provided in the Source Data File.

Supplementary Figure 10

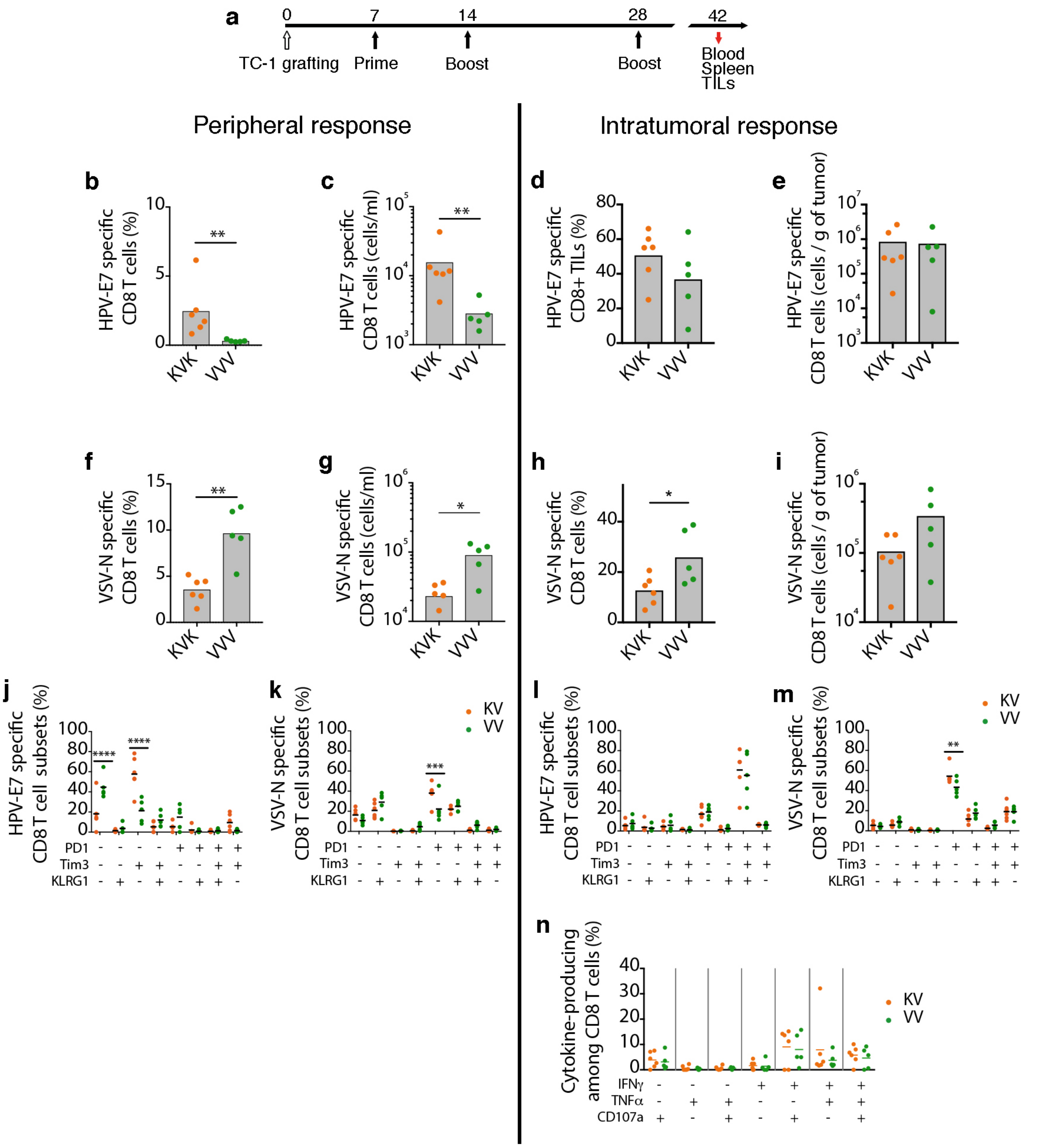

Supplementary Fig. 10. Exhausted intratumoral T cells dominate tumors which relapse after therapeutic vaccination. a-n C57BL/6J mice were injected s.c. with TC-1 cells ($1x10^5$) on day 0 and vaccinated with 2 nmol KISIMA-HPV (s.c.) or $1x10^7$ $TCID_{50}$ VSV-GP-HPV (i.v.) on day 7 and 14. Blood, spleen and tumors were harvested on day 42 for flow cytometric analysis as indicated in schematic **a. b-e** Frequencies and **f-i** numbers of HPV-E7 or VSV-N-specific $CD8^+$ T cells were measured by flow cytometry in **b, c, f, g** blood and in **d, e, h, i** tumors. Two-tailed Mann-Whitney test was performed (*, p<0.05; **, p<0.01). **j-m** Proportions of **j, k** peripheral and **l, m** intratumoral **j, l** HPV- and **k, m** VSV-N specific $CD8^+$ T cells expressing activation and exhaustion markers is shown. 2-way ANOVA with Sidak's multiple comparisons was performed (****, p<0.0001). **n** Fraction of intratumoral HPV-specific $CD8^+$ T cells secreting cytokines upon *ex vivo* restimulation is shown. n = 6 for KVK, n = 5 for VVV. All data shown as individual data points and mean. The study depicted in **b-n** as performed once. Source data and p-values are provided in the Source Data File.

Supplementary Figure 11

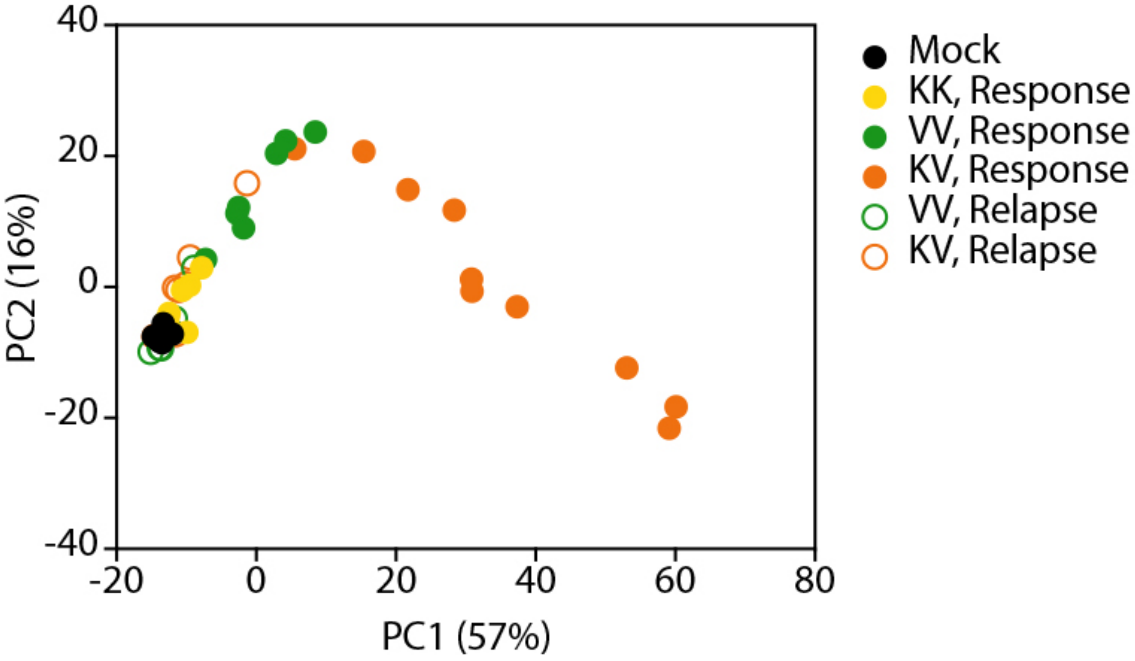

Supplementary Fig. 11. Gene signatures of responding and relapsing TC-1 tumors after different vaccination regimens. C57BL/6J mice were implanted with TC-1 cells ($1x10^5$, s.c.) and immunized s.c. with 2 nmol KISIMA-HPV (K) or i.v. with $1x10^7$ $TCID_{50}$ VSV-GP-HPV (V) on day 7 and 14 post tumor implantation or left untreated (mock). Tumors were harvested during response phase (day 23 post tumor implantation, peak of tumor-specific T cell response) and relapse phase (day 50-60 post tumor implantation) for transcriptome analysis using NanoString® technology. Normalized gene counts for 770 genes included in the NanoString® panel was used to calculate principal component analysis (PCA). The first two components of the PCA is shown. n= 10 for KV treated tumors at response phase and n= 7 for every other treatment group and phase of tumor growth. This study was performed once. Source data are provided in the Source Data File.

Supplementary Figure 12

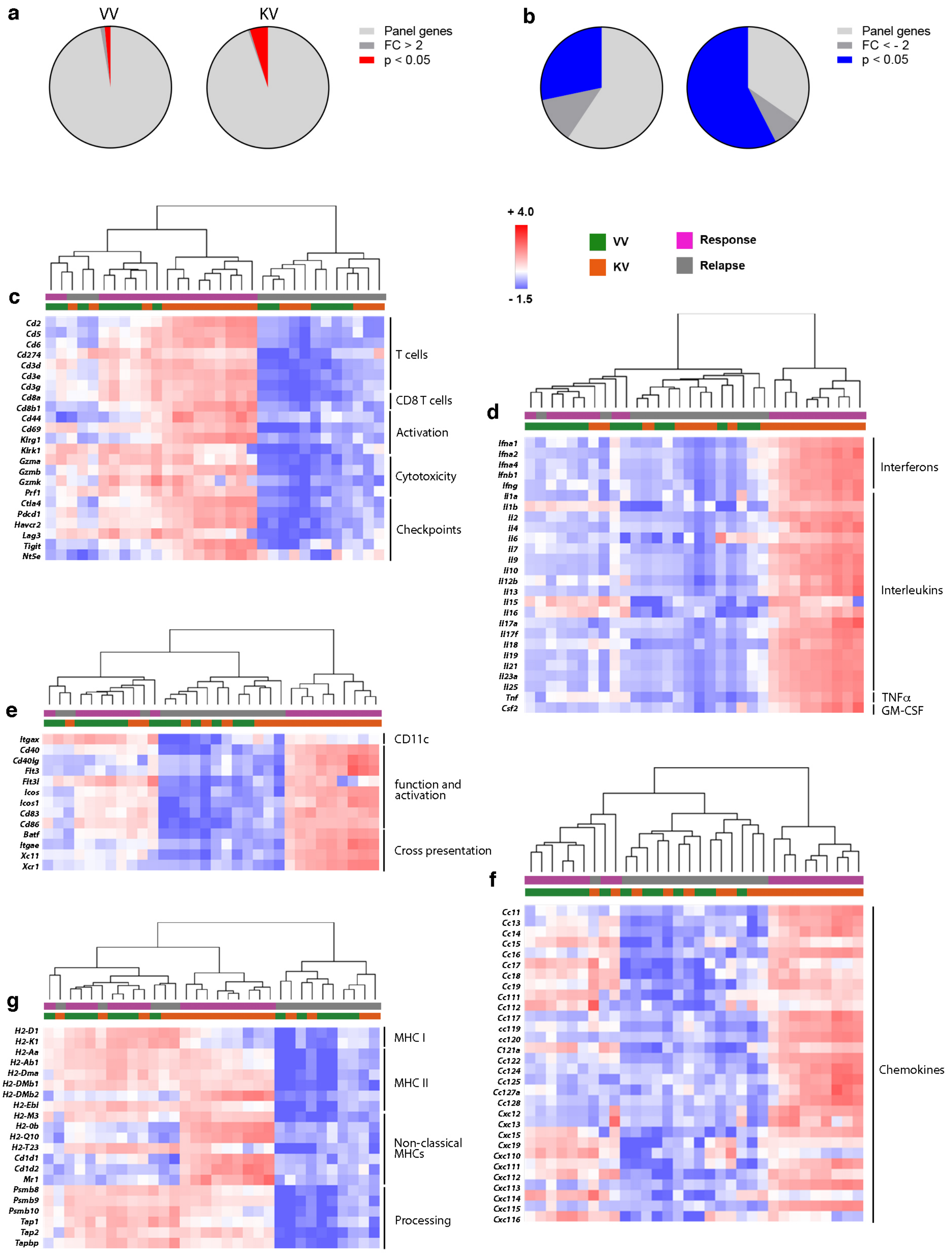

Supplementary Fig. 12. Gene signatures after heterologous vaccination indicate loss of immune activation in relapsing tumors. a-g C57BL/6J mice were implanted with TC-1 cells ($1x10^5$, s.c.) and immunized s.c. with 2 nmol KISIMA-HPV (K) or i.v. with $1x10^7$ $TCID_{50}$ VSV-GP-HPV (V) on day 7 and 14 post tumor implantation. Tumors were harvested during response phase (day 23 post tumor

implantation, peak of tumor-specific T cell response) and relapse phase (day 50-60 post tumor implantation) for transcriptome analysis using Nanostring® technology. n= 10 for KV treated tumors at response phase and n= 7 for every other treatment group and phase of tumor growth. **a, b** Gene expression in TC-1 tumors from relapsing tumors was normalized to responding tumors from the respective vaccination group and the proportion of **a** significantly upregulated (fold change >2, FDR adjusted p value < 0.05) and **b** significantly downregulated (negative reciprocal of fold change < -2 and FDR adjusted p value < 0.05) is displayed. Two-tailed t test and false discovery rate (FDR) adjusted p values calculated using Benjamini-Yekutieli procedure are reported. **c-g** Heatmaps display relative gene expression as z-scores (scaled to each gene) and hierarchical clustering (Euclidean distance, average linkage) was applied to sample data with each column representing one individual tumor. Expression of typical genes associated with **c** cytotoxic T cells, **d** cytokines, **e** dendritic cells, **f** chemokines and **g** antigen presentation is shown. False discovery rate (FDR) adjusted p values were calculated using Benjamini-Yekutieli procedure. The study as shown was performed once. Source data and are provided in the Source Data File.

Supplementary Figure 13

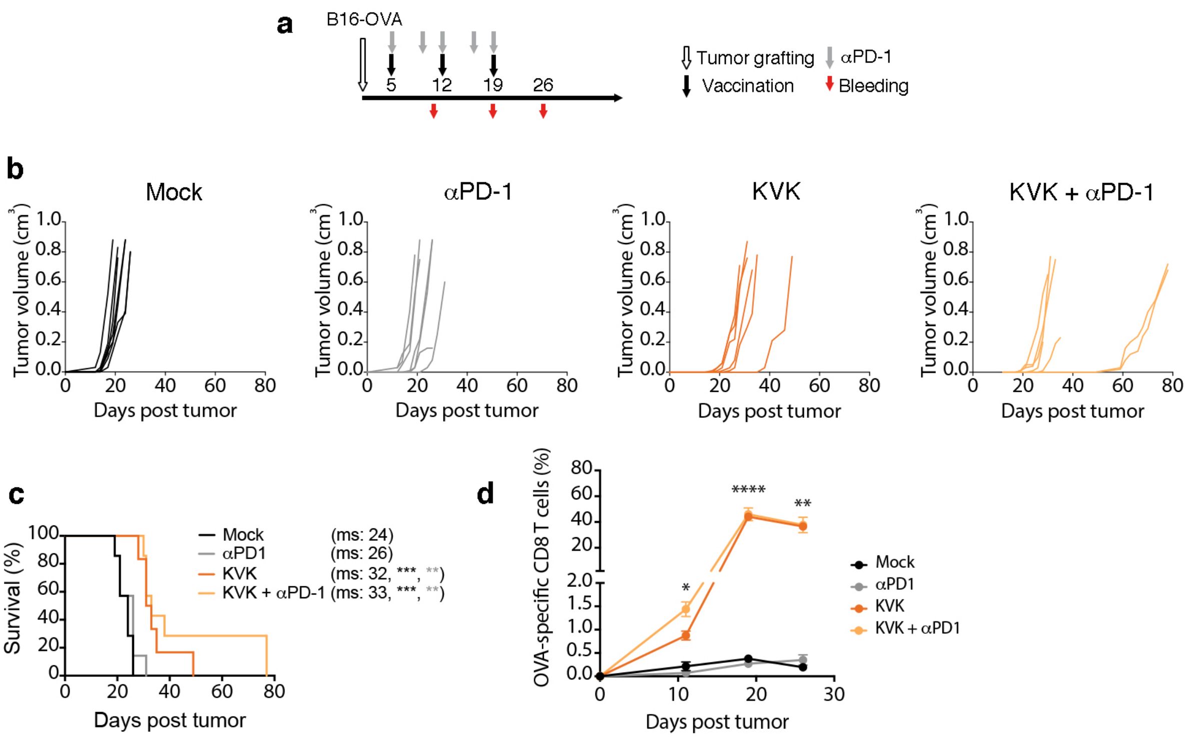

Supplementary Fig. 13. Therapeutic effect of KVK vaccine in syngeneic murine melanoma tumor model expressing ovalbumin. a-d C57BL/6J mice were subcutaneously injected with $1x10^5$ B16-OVA cells (n=5 per treatment group). Mice were treated with KISIMA-OVA (2 nmol, s.c.), VSV-GP-OVA ($1x10^7$ $TCID_{50}$, i.v.) or aPD-1 antibody (200 µg, i.p.) on days indiacted in schematic **a. b** Tumor growth and **c** survival after treatment is depicted. Pairwise Log-rank test was performed (**p<0.01; ***p<0.001). The number of complete responders (red) among total mice (black) is indicated in parentheses beside the tumor growth curves for every treatment group. **d** Frequency of circulating OVA-specific $CD8^+$ T cells in peripheral blood is shown (mean ± SEM). 2-way ANOVA with Tukey's multiple comparison was performed (*p<0.05; **p<0.01; ****p<0.0001). This study was performed once. Source data and p-values are provided in the Source Data File.

Supplementary table 1. Genes uniquely upregulated after KV vaccination. List of genes uniquely upregulated in TC-1 tumors after heterologous KV vaccination. Genes with fold change > 2.0 (normalized to mock treated tumors) and FDR adjusted p value < 0.05 as shown in **Fig. 3c**. Two-tailed t test and false discovery rate (FDR) adjusted p values calculated using Benjamini-Yekutieli procedure are reported.

**Gene**	**Fold change**	**Gene**	**Fold change**	**Gene**	**Fold change**
Cd36	57.35	*Igll1*	25.87	*Tnfrsf17*	13.44
Ifna1	55.69	*Pou2af1*	25.38	*Ambp*	13.04
Il21	50.03	*Cd22*	25.25	*Tnfsf8*	12.99
Cxcl1	49.91	*Cd160*	25.08	*St6gal1*	12.86
Gbp2b	47.13	*Cd8b1*	25	*Cfi*	12.51
Lamp3	46.09	*Ido1*	24.85	*Ms4a1*	12.45
Il25	43.57	*Cxcl15*	24.5	*Ltf*	12.42
Klra7	42.96	*Tnfsf4*	24.14	*Hamp*	12.31
Ccl22	42.69	*Timd4*	23.26	*Il1rapl2*	12.22
Fcer1a	42.53	*Il5ra*	22.94	*Il17rb*	12.19
C8b	42.13	*Cxcl2*	22.63	*Pax5*	12.15
Defb1	42.11	*Cxcr2*	22.48	*Il10*	12.07
H2-Ob	41.66	*C8a*	21.92	*Il1a*	12.02
Il19	41.36	*Klra1*	21.81	*Cdk1*	12.01
Klra3	39.83	*Cd59b*	21.47	*Pmch*	11.91
C9	38.99	*Cd96*	21.32	*Cd163*	11.8
Il2	38.71	*Ifna4*	21.22	*Cr2*	11.74
CD209e	37.28	*Ccl20*	20.98	*Lyve1*	11.47
Chil3	37.24	*Lta*	20.67	*Il13*	11.45
Tdo2	35.57	*Mbl2*	20.62	*Tnfrsf4*	11.38
Eomes	35.02	*Tnfsf15*	20.61	*Itgb3*	11.31
Cxcl5	34.91	*Irf4*	20.49	*Cd5*	11.15
Prg2	34.6	*C6*	19.88	*A2m*	11.14
Pin1	33.94	*Ccr9*	19.14	*Lcn2*	11.04
Ifnb1	32.6	*Ccr3*	17.82	*Epcam*	11.03
Klrb1c	32.36	*Ticam2*	17.07	*Ifng*	10.96
Mnx1	32.22	*Arg2*	16.66	*Tnfrsf13c*	10.93
Ccl1	32.09	*Xcr1*	16.57	*Gata3*	10.78
Slamf1	31.17	*Elk1*	16.32	*Cd1d2*	10.63
Tal1	30.87	*Il9*	16.23	*C7*	10.6
Ifi44l	30.47	*Rorc*	15.55	*S100b*	10.53
Il17f	29.85	*Tnfsf13*	15.43	*Itgae*	10.47
Crp	28.82	*Nos2*	14.59	*Ifna2*	10.38
Tnfsf18	28.67	*Ccr6*	14.46	*Raet1*	10.37
Ppbp	28.13	*Saa1*	13.86	*Il22ra1*	10.3

Il23r	27.74	*Aicda*	13.66	*Cxcr1*	10.21
Klrg1	26.13	*Cd1d1*	13.63	*Trem2*	10.12
Mme	26.12	*Klra6*	13.62	*Cd40lg*	9.68
H2-Q10	26.01	*Sele*	13.6	*Il6*	9.22
Il23a	25.97	*Cxcl13*	13.58	*Ifnl2*	9.04
Trem1	8.96	*Angpt1*	5.85	*Fut7*	2.97
Lif	8.84	*Gpr44*	5.85	*Abca1*	2.95
Ccr4	8.79	*Itga2b*	5.77	*Ikbkb*	2.89
Mpo	8.61	*Ccl25*	5.58	*Irak2*	2.87
Adora2a	8.47	*F12*	5.52	*Mavs*	2.87
Cd207	8.46	*Rag1*	5.23	*Rel*	2.85
Fcer2a	8.37	*Cxcr5*	5.22	*Il12rb1*	2.81
Hras	8.37	*Icam4*	5.16	*Dll4*	2.76
Ctsg	8.09	*Tlr5*	5.1	*Ly96*	2.75
Tnfrsf13b	8	*Mr1*	5.08	*Prkcd*	2.71
Ccl17	7.77	*Bid*	4.89	*Fcgr2b*	2.64
Serpinb2	7.73	*Ccl27a*	4.81	*Il17ra*	2.58
Cdh1	7.64	*Atm*	4.78	*Tank*	2.58
Masp2	7.64	*Cd44*	4.76	*Itch*	2.57
Mst1r	7.63	*Rps6*	4.69	*Mertk*	2.55
Smpd3	7.55	*Atg10*	4.62	*Tbk1*	2.55
Slc7a11	7.39	*Gfi1*	4.62	*Ifnar2*	2.54
Blk	7.38	*Bmi1*	4.6	*Creb5*	2.52
Cd79a	7.33	*Arg1*	4.55	*Tnfsf12*	2.51
Ccl28	7.27	*Tnfaip3*	4.5	*Tirap*	2.5
Ccr1	7.19	*Il17a*	4.43	*Irf2*	2.45
Il12b	7.05	*Tnfrsf11b*	4.15	*Prkce*	2.43
Ceacam1	7.02	*Kit*	4.1	*Smad3*	2.42
Il4	7.01	*Atg5*	3.99	*Ifnar1*	2.38
Cd33	6.96	*Tbx21*	3.91	*Cd14*	2.35
Cxcl3	6.93	*Mef2c*	3.9	*Bcl2*	2.33
C5ar1	6.87	*Tlr4*	3.89	*Hsd11b1*	2.33
Tpsab1	6.86	*Cd3eap*	3.81	*Mcam*	2.31
Csf2	6.8	*Nt5e*	3.7	*Tnfrsf10b*	2.26
Klrb1	6.73	*Vwf*	3.7	*Stat6*	2.23
Osm	6.57	*Ticam1*	3.64	*Itga2*	2.22
Ltk	6.53	*Creb1*	3.55	*Casp8*	2.21
Ccl24	6.42	*Angpt2*	3.51	*Il6ra*	2.21
Pnma1	6.19	*Card9*	3.5	*Cd276*	2.16
F13a1	6.05	*Ripk2*	3.42	*Trp53*	2.15
Flt3	6.05	*Ccnd3*	3.32	*Tubb5*	2.14
Cd68	6	*Irak4*	3.15	*Ep300*	2.12
Chit1	5.96	*Traf3*	3.09	*Jak2*	2.1

Mrc1	5.96	*Ythdf2*	3.04	*Plaur*	2.09
Csf3	5.88	*Atg12*	3.01	*Erbb2*	2.05
Fadd	5.88	*Ikbkg*	2.99	*Cdh5*	2.02

Supplementary table 2. Genes uniquely downregulated after KV vaccination. List of genes uniquely downregulated in TC-1 tumors after heterologous KV vaccination. Genes with negative reciprocal fold change < -2.0 (normalized to mock treated tumors) and FDR adjusted p value < 0.05 as shown in **Fig. 3d**. Two-tailed t test and false discovery rate (FDR) adjusted p values calculated using Benjamini-Yekutieli procedure are reported.

**Gene**	**Fold change**
Msln	-28.02
Ifitm1	-8.81
Itgb4	-7
Tnfrsf12a	-6.98
F2rl1	-6.51
Ptgs2	-6.26
Tfrc	-5.79
Egr1	-5.66
Fez1	-5.58
Cd9	-4.93
Casp3	-4.42
Il1rl1	-4.32
Cdkn1a	-4.05
Cx3cl1	-3.6
Myc	-3.42
Nfatc4	-3.37
Cebpb	-3.34
Thbd	-3.23
Hdac3	-3.2
Itgb1	-3.08
Egr3	-3.01
Lrp1	-2.97
Yy1	-2.8
Jun	-2.72
Dusp6	-2.48
Map3k5	-2.46
Thy1	-2.44
Bax	-2.43
Cd81	-2.39
Birc5	-2.37
Igf2r	-2.17
Ctsl	-2.13
Cd63	-2.07
Prdm1	-2.07
Pvr	-2.03

Supplementary table 3. Rechallenge protection in E.G7-OVA tumor model. Mice bearing E.G7-OVA tumors which were in long-term remission (over 80 days post tumor implanation) as a result of different vaccination regimens were subcutaneously rechallenged with $3x10^5$ E.G7-OVA tumor cells on the contralateral side and proportion of mice that remained tumor-free is depicted.

**Treatment**	**Long-term survivors**	**Protected against rechallenge (%)**
KKKK	1	0
KVKK	1	100
KVKK + αPD-1	4	100
$KV_{\phi}KK$	1	100

Supplementary table 4. Rechallenge protection in MC-38 tumor model. Mice bearing MC-38 tumors which were in long-term remission (over 75 days post tumor implanation) as a result of different vaccination regimens were subcutaneously rechallenged with $2x10^5$ MC-38 tumor cells on the contralateral side and proportion of mice that remained tumor-free is depicted.

**Treatment**	**Long-term survivors**	**Protected against rechallenge (%)**
αPD-L1	1	100
VVVV	4	75
KVKK	1	100
KVKK + αPD-L1	5	100
$KV_{\phi}KK$	1	100
$V_{\phi}V_{\phi}V_{\phi}V_{\phi}$	2	100

Supplementary table 5. Rechallenge protection in TC-1 tumor model. Mice bearing TC-1 tumors which were in long-term remission (over 85 days post tumor implanation) as a result of different vaccination regimens were subcutaneously rechallenged with $1x10^5$ TC-1 tumor cells on the contralateral side and proportion of mice that remained tumor-free is depicted. Data shown are from 3 independent experiments.

**Treatment**	**Long-term survivors**	**Protected against rechallenge (%)**
VVVV	5	60
KVKK	6	100
KVKK + αPD-1	4	75

Supplementary table 6. List of MHC-multimers. List of MHC multimers used for detection of antigen-specific CD8$^+$ T cells after immunization.

**Antigen**	**Haplotype**	**Epitope**	**Supplier**
OVA	H2-Kb	SIINFEKL	MBL International, Woburn, MA, US
Adpgk	H2-Db	ASMTNMELM	MBL International
VSV-N	H2-Kb	RGYVYQGL	MBL International
HPV-E7	H2-Db	RAHYNIVTF	Immudex, Copenhagen, Denmark

Supplementary table 7. Antibodies for characterization of CTLs. List of antibodies for detection and phenotyping of antigen-specific CD8$^+$ T cells induced after immunization.

Antibody	Clone	Dilution used	Supplier
**CD8**	53-6.7	1:200	Biolegend
**CD90.2**	30-H12	1:200	Biolegend
**CD44**	IM7	1:100	Biolegend
**CD62L**	MEL-14	1:100	Biolegend
**CD127**	SB/199	1:50	Biolegend
**KLRG1**	2F1	1:100	Biolegend
**CD4**	GK1.5	1.200	Biolegend
**CD19**	6D5	1:100	Biolegend
**CD14**	Sa14-2	1:100	Biolegend

Supplementary table 8. Antibodies for phenotyping of TILs. List of antibodies for detection and phenotyping of tumor-infiltrating leukocytes.

Antibody	Clone	Dilution used	Supplier
**CD45**	30F11	1:200	BD Biosciences
**CD11b**	M1/70	1:200	BD Biosciences
**CD3**	17A2	1:200	BD Biosciences
**CD4**	RM4-5	1:200	BD Biosciences
**CD8**	53-6.7	1:50	BD Biosciences
**CD25**	3C7	1:200	BD Biosciences
**KLRG1**	2F1	1:50	BD Biosciences
**CD279**	29F.1A12	1:400	Biolegend
**CD366**	RMT3-23	1:100	Biolegend
**Ly6C**	AL-21	1:200	BD Biosciences
**Ly6G**	1A8	1:400	BD Biosciences
**Ly6C/G (Gr-1)**	RB6-8C5	1:200	Biolegend
**CD335**	29A1.4	1:200	BD Biosciences
**CD11c**	HL3	1:100	BD Biosciences
**CD103**	M290	1:200	BD Biosciences
**I-A/I-E**	M5/114.5.2	1:100	BD Biosciences
**FoxP3**	FJK-16s	1:200	eBioscience
**CD206**	C068C2	1:200	Biolegend
**CD68**	FA-11	1:200	BD Biosciences

Supplementary table 9. Antibodies for intracellular staining. List of antibodies for assessing functionality via intracellular cytokine staining of $CD8^+$ T cells.

Antibody	Clone	Dilution used	Supplier
**CD107a**	1D4B	1:200	BD Biosciences
**IFN-γ**	XMG1.2	1:200	BD Biosciences
**TNF-α**	MP6-XT22	1:200	BD Biosciences
**GrzmB**	REA226	1:25	Miltenyi Biotec
