# Supplementary material for: A modular self-adjuvanting cancer vaccine combined with an oncolytic vaccine induces potent antitumor immunity
Source: Nat Commun. 2021 Aug 31;12:5195. doi: 10.1038/s41467-021-25506-6 (PMC8408233; doi:10.1038/s41467-021-25506-6)
Supplement: Supplementary file 3 — Description of Additional Supplementary Files [file 41467_2021_25506_MOESM3_ESM.pdf]

Description of Additional Supplementary Files

File name: Supplementary Data 1.
Description: Gene Expression Profiling of NanoString®nCounter

File name: Supplementary Data 2.
Description: Statistics of Differentially Expressed Genes by NanoString® nCounter analysis during Response Phase

File name: Supplementary Data 3.
Description: Statistics of Differentially Expressed Genes by NanoString® nCounter analysis during Relapse vs Response Phase
