# Supplementary material for: A modular self-adjuvanting cancer vaccine combined with an oncolytic vaccine induces potent antitumor immunity
Source: Nat Commun. 2021 Aug 31;12:5195. doi: 10.1038/s41467-021-25506-6 (PMC8408233; doi:10.1038/s41467-021-25506-6)
Supplement: Supplementary file 7 — Reporting Summary [file 41467_2021_25506_MOESM7_ESM.pdf]

nature research

Corresponding author(s): Guido Wollmann, Madiha Derouazi

Last updated by author(s): May 30, 2021

Reporting Summary

Nature Research wishes to improve the reproducibility of the work that we publish. This form provides structure for consistency and transparency in reporting. For further information on Nature Research policies, see Authors & Referees and the Editorial Policy Checklist.

Statistics

For all statistical analyses, confirm that the following items are present in the figure legend, table legend, main text, or Methods section.

n/a	Confirmed	
☐	☒	The exact sample size (*n*) for each experimental group/condition, given as a discrete number and unit of measurement
☐	☒	A statement on whether measurements were taken from distinct samples or whether the same sample was measured repeatedly
☐	☒	The statistical test(s) used AND whether they are one- or two-sided<br>*Only common tests should be described solely by name; describe more complex techniques in the Methods section.*
☒	☐	A description of all covariates tested
☐	☒	A description of any assumptions or corrections, such as tests of normality and adjustment for multiple comparisons
☐	☒	A full description of the statistical parameters including central tendency (e.g. means) or other basic estimates (e.g. regression coefficient) AND variation (e.g. standard deviation) or associated estimates of uncertainty (e.g. confidence intervals)
☐	☒	For null hypothesis testing, the test statistic (e.g. F, t, r) with confidence intervals, effect sizes, degrees of freedom and P value noted<br>*Give P values as exact values whenever suitable.*
☒	☐	For Bayesian analysis, information on the choice of priors and Markov chain Monte Carlo settings
☒	☐	For hierarchical and complex designs, identification of the appropriate level for tests and full reporting of outcomes
☒	☐	Estimates of effect sizes (e.g. Cohen's d, Pearson's r), indicating how they were calculated

Our web collection on statistics for biologists contains articles on many of the points above.

Software and code

Policy information about availability of computer code

Data collection	Lumina system (IVIS Lumina II, Perkin Elmer, Waltham, Massachusetts, USA)<br>nCounter FLEX Analysis System (NanoString Technologies, Seattle, WA, USA)<br>Olympus BX-53 microscope (Olympus, Tokyo, Japan)<br>FACS Canto II (BD Biosciences, San Jose, California, US)<br>Gallios flow cytometer (Beckman Coulter, Brea, California, US)<br>Attune NxT (ThermoFisher, Waltham, Massachusetts, United States)<br>Epoch Microplate Spectrophotometer (BioTek Instruments Inc., Winooski, Vermont, USA)<br>Tapestation 4200 (Agilent Technologies, Waldbronn, Germany)
Data analysis	Prism 8 (Graphpad, San Diego, California, US)<br>nSolver 4.0 software (NanoString Technologies, Seattle, WA, USA)<br>ClustVis (https://biit.cs.ut.ee/clustvis/)<br>Venn diagram webtool (http://bioinformatics.psb.ugent.be/webtools/Venn/)<br>LEGENDplex™ Cloud-based Data Analysis Software (BioLegend, San Diego, California, US)<br>FlowJo software version 10.5.3 (FlowJo, LLC, Oregon, US)<br>Kaluza software 2.1 (Beckman Coulter, Brea, California, US)<br>Figure generation and processing: Adobe Photoshop CS6 (Adobe, Mountain View, California, US)

For manuscripts utilizing custom algorithms or software that are central to the research but not yet described in published literature, software must be made available to editors/reviewers. We strongly encourage code deposition in a community repository (e.g. GitHub). See the Nature Research guidelines for submitting code & software for further information.

Data

Policy information about availability of data

All manuscripts must include a data availability statement. This statement should provide the following information, where applicable:

- Accession codes, unique identifiers, or web links for publicly available datasets
- A list of figures that have associated raw data
- A description of any restrictions on data availability

The authors confirm that the pertinent data supporting the findings of this study are included in the article and the supplementary materials.

Field-specific reporting

Please select the one below that is the best fit for your research. If you are not sure, read the appropriate sections before making your selection.

☒ Life sciences ☐ Behavioural & social sciences ☐ Ecological, evolutionary & environmental sciences

For a reference copy of the document with all sections, see nature.com/documents/nr-reporting-summary-flat.pdf

Life sciences study design

All studies must disclose on these points even when the disclosure is negative.

Sample size	Sample sizes were chosen empirically based on experience from our previous studies using the presented or similar tumor models with (PMID: 29498639, 30972741, 31530903) or without (PMID: 27377043, 31013258) oncolytic virotherapies, which were found to meet estimated statistical power. The "3R's" were adhered to. Reporting according to ARRIVE guidlines was applied.
Data exclusions	No experimental data were excluded after initiation of treatments.
Replication	In-vivo experiments were repeated in some cases and details are listed in the figure legends. Efficacy of heterologous prime-boost in TC-1 tumor model was repeated 3 times and a representative experiment is shown in Fig 6. The animals in long-term remission from the three experiments were rechallenged with TC-1 tumor cells and proportion of protected mice is reported in supplementary table 5.
Randomization	For immunization in non-tumor bearing animals, mice were randomly assigned to different treatment groups. Mice bearing TC-1 tumors were grouped based on tumor size prior to first immunization on day 7 post tumor implantation so that the mean tumor size of each treatment group was comparable. In case of mice bearing E.G7-OVA, B16-OVA or MC-38 tumors, mice were randomly assigned to different treatment groups after tumor implantation and before treatment start to reduce any variability arising during cell preparation and tumor implantation.
Blinding	For most parts, investigators were not blinded during data collection or analysis because planning, execution, and analysis of the studies was performed by the same personnel. Histological analysis of the immunohistochemistry sections by the pathologist as well as the processing of the samples for nanostring transcriptom analysis was blinded.

Reporting for specific materials, systems and methods

We require information from authors about some types of materials, experimental systems and methods used in many studies. Here, indicate whether each material, system or method listed is relevant to your study. If you are not sure if a list item applies to your research, read the appropriate section before selecting a response.

Materials & experimental systems

n/a	Involved in the study	
☐	☒	Antibodies
☐	☒	Eukaryotic cell lines
☒	☐	Palaeontology
☐	☒	Animals and other organisms
☒	☐	Human research participants
☒	☐	Clinical data

Methods

n/a	Involved in the study	
☒	☐	ChIP-seq
☐	☒	Flow cytometry
☒	☐	MRI-based neuroimaging

Antibodies

Antibodies used	Immunohistochemistry:<br>Primary monoclonal antibody against CD8α (clone D4W2Z, 1:2000, #98941, Cell Signaling, Danvers, Massachusetts, US)<br>Secondary antibody conjugated to horse radish peroxidase (ready to use, #DPVR110HRP, ImmunoLogic, Duiven, Netherlands)<br>Flow cytometry<br>CD8α (Clone 53-6.7, 1:200, #100752, Biolegend, San Diego, California, US)<br>CD90.2 (Clone 30-H12, 1:200, #105316, Biolegend)

CD44 (Clone IM7, 1:100, #103040, Biolegend)
CD62L (Clone MEL-14, 1:100, #104418, Biolegend)
CD127 (Clone SB/199, 1:50, #121114, Biolegend)
KLRG1 (Clone 2F1, 1:100, #138416, Biolegend)
CD4 (Clone GK1.5, 1:200, #100414, Biolegend)
CD19 (Clone 6D5, 1:100, #115530, Biolegend)
CD14 (Clone Sa14-2, 1:100, #123317, Biolegend)
CD45 (Clone 30F11, 1:200, #563890, BD Biosciences, San Jose, California, US)
CD11b (Clone M1/70, 1:200, #563168, BD Biosciences)
CD3 (Clone 17A2, 1:200, #560527, BD Biosciences)
CD4 (Clone RM4-5, 1:200, #565634, BD Biosciences)
CD8 (Clone 53-6.7, 1:50, #560182, BD Biosciences)
CD25 (Clone PC61.5, 1:200, #45-0251-82, eBiosciences, San Diego, California, US)
KLRG1 (Clone 2F1, 1:50, #562897, BD Biosciences)
CD279 (Clone 29F.1A12, 1:400, #135216, Biolegend)
CD366 (Clone RMT3-23, 1:100, #119718, Biolegend)
Ly6C (Clone AL-21, 1:200, #560595, BD Biosciences)
Ly6G (Clone 1A8, 1:400, #562737, BD Biosciences)
Gr-1 (Clone RB6-8C5, 1:200, #562709, BD Biosciences)
CD335 (Clone 29A1.4, 1:200, #560757, BD Biosciences)
CD11c (Clone HL3, 1:100, #562782, BD Biosciences)
CD103 (Clone M290, 1:200, #564320, BD Biosciences)
I-A/I-E (Clone M5/114.5.2, 1:100, #562564, BD Biosciences)
FoxP3 (Clone FJK-16s, 1:200, #12-5773-82, eBioscience)
CD206 (Clone C068C2, 1:200, #141708, Biolegend)
CD68 (Clone FA-11, 1:200, #137024, Biolegend)
CD107a (Clone 1D4B, 1:200, #564348, BD Biosciences)
IFN-γ (Clone XMG1.2, 1:200, #554413, BD Biosciences)
TNF-α (Clone MP6-XT22, 1:200, #554419, BD Biosciences)
GrzmB (Clone REA226, 1:25, #130-120-703, Miltenyi Biotec, Bergisch Gladbach, Germany)

Validation

The following lists application or validation references for antibodies from the respective antibody manufacturers and or suppliers:
CD8α: Saha D, Martuza RL, Rabkin SD. Macrophage Polarization Contributes to Glioblastoma Eradication by Combination Immunovirotherapy and Immune Checkpoint Blockade. Cancer Cell. 2017;32(2):253-267.e5. doi:10.1016/j.ccell.2017.07.006. PMID: 28810147.
CD90.2: Armaka M, Ospelt C, Pasparakis M, Kollias G. The p55TNFR-IKK2-Ripk3 axis orchestrates arthritis by regulating death and inflammatory pathways in synovial fibroblasts. Nat Commun. 2018;9(1):618. Published 2018 Feb 12. doi:10.1038/s41467-018-02935-4. PMID: 29434332.
CD44: Findlay EG, Currie AJ, Zhang A, et al. Exposure to the antimicrobial peptide LL-37 produces dendritic cells optimized for immunotherapy. Oncoimmunology. 2019;8(8):1608106. Published 2019 May 1. doi:10.1080/2162402X.2019.1608106. PMID: 31413918.
CD62L: LaFleur MW, Nguyen TH, Coxe MA, et al. A CRISPR-Cas9 delivery system for in vivo screening of genes in the immune system. Nat Commun. 2019;10(1):1668. Published 2019 Apr 10. doi:10.1038/s41467-019-09656-2. PMID: 30971695.
CD127: Maeda K, Baba Y, Nagai Y, et al. IL-6 blocks a discrete early step in lymphopoiesis. Blood. 2005;106(3):879-885. doi:10.1182/blood-2005-02-0456. PMID: 15831701.
KLRG1: Herndler-Brandstetter D, Ishigame H, Shinnakasu R, et al. KLRG1+ Effector CD8+ T Cells Lose KLRG1, Differentiate into All Memory T Cell Lineages, and Convey Enhanced Protective Immunity. Immunity. 2018;48(4):716-729.e8. doi:10.1016/j.immuni.2018.03.015. PMID: 29625895.
CD4: Ershaid N, Sharon Y, Doron H, et al. NLRP3 inflammasome in fibroblasts links tissue damage with inflammation in breast cancer progression and metastasis. Nat Commun. 2019;10(1):4375. Published 2019 Sep 26. doi:10.1038/s41467-019-12370-8. PMID: 31558756.
CD19: Stone AEL, Green R, Wilkins C, Hemann EA, Gale M Jr. RIG-I-like receptors direct inflammatory macrophage polarization against West Nile virus infection. Nat Commun. 2019;10(1):3649. Published 2019 Aug 13. doi:10.1038/s41467-019-11250-5. PMID: 31409781.
CD14: Jeljeli M, Riccio LGC, Doridot L, et al. Trained immunity modulates inflammation-induced fibrosis. Nat Commun. 2019;10(1):5670. Published 2019 Dec 11. doi:10.1038/s41467-019-13636-x. PMID: 31827093.
CD45: Johnson P, Maiti A, Ng DHW. CD45: A family of leukocyte-specific cell surface glycoproteins. In: Herzenberg LA, Weir DM, Herzenberg LA, Blackwell C , ed. Weir's Handbook of Experimental Immunology, Vol 2. Cambridge: Blackwell Science; 1997; :62.1-62.16.
CD11b: Ault KA, Springer TA. Cross-reaction of a rat-anti-mouse phagocyte-specific monoclonal antibody (anti-Mac-1) with human monocytes and natural killer cells. J Immunol. 1981; 126(1):359-364.
CD3: Miescher GC, Schreyer M, MacDonald HR. Production and characterization of a rat monoclonal antibody against the murine CD3 molecular complex. Immunol Lett. 1989; 23(2):113-118.
CD4: Bendelac A. Mouse NK1+ T cells. Curr Opin Immunol. 1995; 7(3):367-374.
CD8: Bierer BE, Sleckman BP, Ratnofsky SE, Burakoff SJ. The biologic roles of CD2, CD4, and CD8 in T-cell activation. Annu Rev Immunol. 1989; 7:579-599. (Biology: Flow cytometry).
CD25: Park SG, Mathur R, Long M, Hosh N, Hao L, Hayden MS, Ghosh S. T regulatory cells maintain intestinal homeostasis by suppressing γδ T cells. Immunity. 2010 Nov 24;33(5):791-803. doi: 10.1016/j.immuni.2010.10.014. Epub 2010 Nov 11. PMID: 21074460; PMCID: PMC2996054.
KLRG1: Beyersdorf NB, Ding X, Karp K, Hanke T. Expression of inhibitory "killer cell lectin-like receptor G1" identifies unique subpopulations of effector and memory CD8 T cells. Eur J Immunol. 2001; 31(12):3443-3452.
CD279: Saumyaa, Arjunaraja S, Pujanauski L, Colino J, Torres RM, Snapper CM. Immunosuppressive property within the Streptococcus pneumoniae cell wall that inhibits generation of T follicular helper, germinal center, and plasma cell response to a

coimmunized heterologous protein. Infect Immun. 2013 Sep;81(9):3426-33. doi: 10.1128/IAI.00688-13. Epub 2013 Jul 1. PMID: 23817619; PMCID: PMC3754212.
CD366: Tomaru U, Konno S, Miyajima S, Kimoto R, Onodera M, Kiuchi S, Murata S, Ishizu A, Kasahara M. Restricted Expression of the Thymoproteasome Is Required for Thymic Selection and Peripheral Homeostasis of CD8+ T Cells. Cell Rep. 2019 Jan 15;26(3):639-651.e2. doi: 10.1016/j.celrep.2018.12.078. PMID: 30650357.
Ly6C: Cerwenka A, Carter LL, Reome JB, Swain SL, Dutton RW. In vivo persistence of CD8 polarized T cell subsets producing type 1 or type 2 cytokines. J Immunol. 1998; 161(1):97-105.
Ly6G: Fleming TJ, Fleming ML, Malek TR. Selective expression of Ly-6G on myeloid lineage cells in mouse bone marrow. RB6-8C5 mAb to granulocyte-differentiation antigen (Gr-1) detects members of the Ly-6 family. J Immunol. 1993; 151(5):2399-2408.
Gr-1: Brummer E, Sugar AM, Stevens DA. Immunological activation of polymorphonuclear neutrophils for fungal killing: studies with murine cells and blastomyces dermatitidis in vitro. J Leukoc Biol. 1984; 36(4):505-520.
CD335: Biassoni R, Pessino A, Bottino C, Pende D, Moretta L, Moretta A. The murine homologue of the human NKp46, a triggering receptor involved in the induction of natural cytotoxicity. Eur J Immunol. 1999; 29(3):1014-1020.
CD11c: Burt BM, Plitas G, Stableford JA, et al. CD11c identifies a subset of murine liver natural killer cells that responds to adenoviral hepatitis. J Leukoc Biol. 2008; 84(4):1039-1046.
CD103: Andrew DP, Rott LS, Kilshaw PJ, Butcher EC. Distribution of alpha 4 beta 7 and alpha E beta 7 integrins on thymocytes, intestinal epithelial lymphocytes and peripheral lymphocytes. Eur J Immunol. 1996; 26(4):897-905.
I-A/I-E: Bhattacharya A, Dorf ME, Springer TA. A shared alloantigenic determinant on Ia antigens encoded by the I-A and I-E subregions: evidence for I region gene duplication. J Immunol. 1981; 127(6):2488-2495.
FoxP3: Pang P, Zheng K, Wu S, Xu H, Deng L, Shi Y, Chen X. Baicalin Downregulates RLRs Signaling Pathway to Control Influenza A Virus Infection and Improve the Prognosis. Evid Based Complement Alternat Med. 2018 Feb 26;2018:4923062. doi: 10.1155/2018/4923062. PMID: 29681974; PMCID: PMC5846362.
CD206: He H, Xu J, Warren CM, Duan D, Li X, Wu L, Iruela-Arispe ML. Endothelial cells provide an instructive niche for the differentiation and functional polarization of M2-like macrophages. Blood. 2012 Oct 11;120(15):3152-62. doi: 10.1182/blood-2012-04-422758. Epub 2012 Aug 23. PMID: 22919031; PMCID: PMC3471522.
CD68: Kayama H, Ueda Y, Sawa Y, Jeon SG, Ma JS, Okumura R, Kubo A, Ishii M, Okazaki T, Murakami M, Yamamoto M, Yagita H, Takeda K. Intestinal CX3C chemokine receptor 1(high) (CX3CR1(high)) myeloid cells prevent T-cell-dependent colitis. Proc Natl Acad Sci U S A. 2012 Mar 27;109(13):5010-5. doi: 10.1073/pnas.1114931109. Epub 2012 Mar 7. PMID: 22403066; PMCID: PMC3323962.
CD107a: Arterburn LM, Earles BJ, August JT. The disulfide structure of mouse lysosome-associated membrane protein 1. J Biol Chem. 1990; 265(13):7419-7423.
IFN-γ: Abrams JS, Roncarolo MG, Yssel H, Andersson U, Gleich GJ, Silver JE. Strategies of anti-cytokine monoclonal antibody development: immunoassay of IL-10 and IL-5 in clinical samples. Immunol Rev. 1992; 127:5-24.
TNF-α: Abrams JS, Roncarolo MG, Yssel H, Andersson U, Gleich GJ, Silver JE. Strategies of anti-cytokine monoclonal antibody development: immunoassay of IL-10 and IL-5 in clinical samples. Immunol Rev. 1992; 127:5-24.
GrzmB: Grossman, W. J. et al. (2004) Differential expression of granzymes A and B in human cytotoxic lymphocyte subsets and T regulatory cells. Blood 104: 2840-2848

Eukaryotic cell lines

Policy information about cell lines

Cell line source(s)	E.G7-OVA cells (American Type Culture Collection (ATCC), Manassas, VA)<br>The following cell lines were not obtained from commercial vendors but from academic institutions:<br>TC-1 cells (T.C. Wu, Johns Hopkins University, Maryland, US)<br>MC-38 cells (Gottfried Baier, Medical University of Innsbruck, Innsbruck, Austria)<br>B16-OVA cells (Bertrand Huard, University of Grenoble-Alpes, Grenoble, France)
Authentication	TC-1 cells were validated for the expression of E6 and E7 RNA by performing RT-PCR followed by sequencing of the amplicons. Similarly, MC-38 cells were validated for the expression of the neoepitopes Adpgk and Reps1 by RT-PCR followed by sequencing of the amplicons. Genomic authentication was not performed for B16-OVA and E.G7-OVA.
Mycoplasma contamination	All cell lines were tested negative for mycoplasma contamination.
Commonly misidentified lines<br>(See ICLAC register)	None of the cell lines used in this study were commonly misidentified lines listed in ICLAC.

Animals and other organisms

Policy information about studies involving animals; ARRIVE guidelines recommended for reporting animal research

Laboratory animals	Six- to eight-week old female C57BL/6Rj or B6(C)/Rj-Tyrc/c (albino) mice were used and housed in a BL2 facility in individually ventilated cages with a 12-hour light/dark cycle with unrestricted access to food and water. Temperature in animal facilities was 20-24 °C and humidity was 55 ± 10%. Mice were purchased from Janvier Labs (Le Genest St Isle, France) or Charles River (C57Bl/6Rj stock number 632; L'Arbresles, France).
Wild animals	No wild animals were used in this study.
Field-collected samples	No field-collected samples were used in this study.
Ethics oversight	All animal experiments were approved by Austrian Federal Ministry of Science, Research and Economy and by institutional and cantonal Geneva veterinary authorities in accordance with Swiss Federal law on animal protection and performed according to

institutional guidelines of the Medical University of Innsbruck, Austria and of Swiss Federal law on animal protection, respectively.

Note that full information on the approval of the study protocol must also be provided in the manuscript.

Flow Cytometry

Plots

Confirm that:

☒ The axis labels state the marker and fluorochrome used (e.g. CD4-FITC).

☒ The axis scales are clearly visible. Include numbers along axes only for bottom left plot of group (a 'group' is an analysis of identical markers).

☒ All plots are contour plots with outliers or pseudocolor plots.

☒ A numerical value for number of cells or percentage (with statistics) is provided.

Methodology

Sample preparation

Single cell suspensions from spleen and bone marrow were prepared by mechanical dissociation using a 40 µM cell strainer followed by lysis of erythrocyte using Pharm Lyse Lysing buffer (BD Biosciences, San Jose, California, US). For whole blood, lysis was carried out after surface staining.
Tumor-infiltrating leucocytes (TILs) were purified using mouse tumor dissociation kit (Miltenyi Biotec, Bergisch Gladbach, Germany) and Gentle MACS with heating system (Miltenyi Biotec, Bergisch Gladbach, Germany), following manufacturer instruction. Briefly, tumor tissues were chopped into small pieces, and resuspended in DMEM medium containing tumor dissociating enzymes (Miltenyi Biotec, Bergisch Gladbach, Germany). Tumors were digested on a Gentle MACS with heating system (Miltenyi Biotec, Bergisch Gladbach, Germany) using solid tumor program. Enzymatic digestion was stopped by adding cold PBS 0.5% BSA solution and keeping cells on ice. Digested tumors were passed through a 70µm cell strainer to eliminate remaining undigested tissue. CD45+ cells were purified using CD45 TIL microbeads (Miltenyi Biotec, Bergisch Gladbach, Germany) following manufacturer protocol. Purified CD45+ cells were used for flow cytometry staining or ex vivo T cells stimulation.
For the detection of antigen-specific T cells, whole blood or single cell suspensions from spleen, bone marrow or tumor were incubated with the relevant MHC multimer for 20 minutes at room temperature. The cells were then directly incubated with antibody mix against surface markers for 30 minutes and 4 °C. The cells were then washed twice with PBS and dead cells were labelled with LIVE/DEAD yellow or aqua fluorescent reactive dye from Life Technologies for 30 minutes at 4 °C. The cells were then washed twice with FACS buffer (PBS, 2% FCS, 5mM EDTA). The cells were fixed in FACS buffer containing 1.5% paraformaldehyde for 20 minutes and washed twice with FACS buffer before analysis.
For intracellular cytokine staining, cells were stimulated with HPV-E7 peptide and in presence of CD107a mAb for 6h in CTL medium containing Brefeldin A (GolgiPlug, BD Biosciences), following manufacturer instruction. Then, cells were stained for extracellular markers, fixation and permeabilization was carried out using the BD Bioscience kit according to manufacturer's instructions, and intracellular staining was performed with mAbs to IFN-γ, TNF-α or corresponding isotype controls.
Prior granzyme B intracellular staining, cells were cultured for 4h in the presence of Brefeldin A (GolgiPlug, BD Bioscences). Then, cells were stained for extracellular markers, fixation and permeabilization was carried out using the BD Bioscience kit according to manufacturer's instructions, and intracellular staining was performed with mAb to granzyme B.

Instrument

FACS Canto II (BD Biosciences, San Jose, California, US)
Gallios flow cytometer (Beckman Coulter, Brea, California, US)
Attune NxT (ThermoFisher, Waltham, Massachusetts, United States)

Software

FlowJo software version 10.5.3 (FlowJo, LLC, Oregon, US)
Kaluza software 2.1 (Beckman Coulter, Brea, California, US)

Cell population abundance

The purity of MACS sorted CD45+ tumor infiltrating leukocytes was measured by flow cytometry and ranged between 67 and 97% depending on the sample. Cell population abundance was not precisely evaluated right after sorting because CD45+ selection was used only to increase their frequency, thus facilitating the flow cytometry staining and analysis of tumor infiltrating leukocytes.

Gating strategy

1. Analysis of effector and memory precursors among Ova-specific CD8+ T cells

Cells: SSC-A/FSC-A
Single cells: FSC-W/FSC-A
Viable cells: dead cell marker negative
CD8+ T cells: CD45 positive and CD8 positive
antigen-specific CD8+ T cells: multimer positive
Effector: KLRG1 positive and CD127 negative
Memory precursors: CD127 positive

2. Analysis of effector and central memory cells among E7-specific CD8+ T cells in blood, bone marrow and spleen
Cells: SSC-A/FSC-A
Single cells: FSC-A/FSC-H
Viable and lineage negative: Lineage (CD4, CD19, CD14) negative and dead cell marker negative
CD8+ T cells: CD90.2 positive and CD8 positive

antigen-specific CD8+ T cells: multimer positive
Central memory: CD62L positive and CD44 positive
Effector memory: CD62L negative and CD44 positive
3. Analysis of antigen-specific cells expressing PD-1, Tim-3 and KLRG1 in periphery

Cells: SSC-A/FSC-A
Single cells: FSC-W/FSC-A
Viable cells: dead cell marker negative
lineage negative cells: CD11b, CD4, CD19 negative
antigen-specific CD8+ T cells: CD8 positive and multimer positive
PD-1 and/or Tim-3 and/or KLRG1 positive

4. Analysis of antigen-specific cells expressing PD-1, Tim-3 and KLRG1 in tumor

Cells: SSC-A/FSC-A
Single cells: FSC-W/FSC-A
Viable cells: dead cell marker negative
Non-myeloid cells: CD45 positive and CD11b negative
antigen-specific CD8+ T cells: CD8 positive and multimer positive
PD-1 and/or Tim-3 and/or KLRG1 positive

5. Analysis of cytokine secreting CD8 T cells

Cells: SSC-A/FSC-A
Single cells: FSC-W/FSC-A
Viable cells: dead cell marker negative
CD8+ T cells: CD8 positive and CD4 negative
IFN-⍰ and/or TNF⍰ and/or CD107⍰ positive

6. Analysis of GrzmB positive cells

Cells: SSC-A/FSC-A
Single cells: FSC-W/FSC-A
Viable cells: dead cell marker negative
Non-myeloid cells: CD45 positive and CD11b-
CD8+ T cells: CD8 positive and CD4 negative
Granzyme B producing CD8+ T cells: GzB positive

7. Anaylsis of different subsets of tumor-infiltrating leukocytes

Cells: SSC-A/FSC-A
Single cells: FSC-W/FSC-A
Viable cells: dead cell marker negative
CD8 T cells:
• CD45 positive and CD11b negative
• CD8 positive
CD4 T cells:
• CD45 positive and CD11b negative
• CD4 positive and CD8 negative
• Treg: CD25 positive and Foxp3 positive
• Teff: CD25 negative and Foxp3 negative
TAM:
• CD45 positive and CD11b positive
• Gr-1 negative and CD68 positive
• TAM-1: CD206 negative
• TAM-2 CD206 positive
NK and NKT:
• CD45 positive and CD11b negative
• NK: NKp46 positive and CD3 negative
• NKT: NKp46 positive and CD3 positive
mMDSC:
• CD45 positive and CD11b positive
• mMDSC: Ly6C hi and Ly6G negative
gMDSC:
• CD45 positive and CD11b positive
• Ly6C intermediate and Ly6G intermediate
• SSC low and FSC low
Neutrophils:
• CD45 positive and CD11b positive
• Ly6C intermediate and Ly6G intermediate
• SSC hi and FSC hi
DCs:
• CD45 positive and CD11b positive
• CD11c hi and MHC-II hi
B cells:

• CD45 positive and CD11b negative
• B220 positive and CD11c negative

☒ Tick this box to confirm that a figure exemplifying the gating strategy is provided in the Supplementary Information.
